# Supplementary material for: TGFβ-Smad3 signaling restores cell-autonomous Srsf1-mediated splicing of fibronectin in aged skeletal muscle stem cells
Source: Nat Commun. 2025 Nov 21;16:11532. doi: 10.1038/s41467-025-66582-2 (PMC12748718; doi:10.1038/s41467-025-66582-2)
Supplement: Supplementary file 1 — Supplementary Information [file 41467_2025_66582_MOESM1_ESM.pdf]

Supplementary Information

TGFβ-Smad3 Signaling Restores Cell-Autonomous Srsf1-Mediated Splicing of Fibronectin in Aged Skeletal Muscle Stem Cells

Yuguo Liu, Svenja C. Schüler, Simon Dumontier, Frederic Balg, Sonia Bedard, Thibaut Desgeorges, Jerome N. Feige, Pierre-Luc Boudreault, C. Florian Bentzinger

Supplementary Figures 1-8

Supplementary Tables 1-5

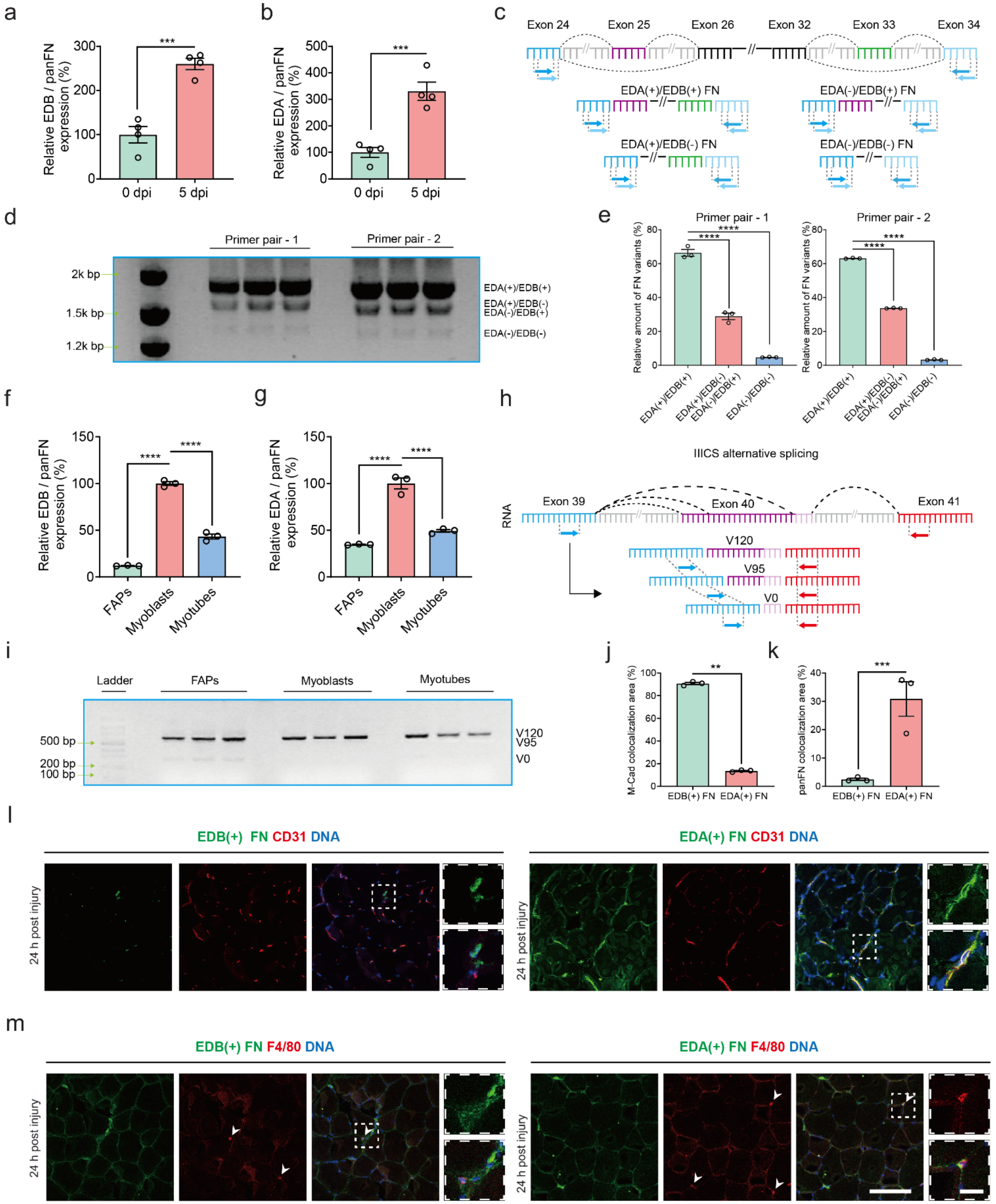
a
Relative EDB / panFN expression (%)
300
200
100
0
***
0 dpi
5 dpi
b
Relative EDA / panFN expression (%)
500
400
300
200
100
0
***
0 dpi
5 dpi
c
Exon 24
Exon 25
Exon 26
Exon 32
Exon 33
Exon 34
EDA(+)/EDB(+) FN
EDA(-)/EDB(+) FN
EDA(+)/EDB(-) FN
EDA(-)/EDB(-) FN
d
Primer pair - 1
Primer pair - 2
2k bp
1.5k bp
1.2k bp
EDA(+)/EDB(+)
EDA(+)/EDB(-)
EDA(-)/EDB(+)
EDA(-)/EDB(-)
e
Primer pair - 1
Primer pair - 2
Relative amount of FN variants (%)
80
60
40
20
0
****
EDA(+)/EDB(+)
EDA(+)/EDB(-)
EDA(-)/EDB(+)
EDA(-)/EDB(-)
f
Relative EDB / panFN expression (%)
150
100
50
0
****
FAPs
Myoblasts
Myotubes
g
Relative EDA / panFN expression (%)
150
100
50
0
****
FAPs
Myoblasts
Myotubes
h
IIICS alternative splicing
RNA
Exon 39
Exon 40
Exon 41
V120
V95
V0
i
Ladder
FAPs
Myoblasts
Myotubes
500 bp
200 bp
100 bp
V120
V95
V0
j
M-Cad colocalization area (%)
100
80
60
40
20
0
**
EDB(+) FN
EDA(+) FN
k
panFN colocalization area (%)
40
30
20
10
0
***
EDB(+) FN
EDA(+) FN
l
EDB(+) FN CD31 DNA
EDA(+) FN CD31 DNA
24 h post injury
m
EDB(+) FN F4/80 DNA
EDA(+) FN F4/80 DNA
24 h post injury

Supplementary Fig. 1: FN splicing during skeletal muscle regeneration.

a,b EDB(+) and EDA(+) FN expression analysis in resting (0 dpi, a) and 5 dpi (b) mouse *tibialis anterior* (*TA*) muscles by quantitative PCR. Data was normalized to total FN (panFN) expression. **c,** Illustration showing alternative splicing of the mouse FN pre-mRNA with the EDB (exon 25) and EDA (exon 33) extra domains as well as the location of primers used for simultaneous PCR detection of both exons. **d,e,** PCR analysis with the primers described in (c) and quantification of the relative amount of EDA and EDB inclusion in mRNA of MuSC-derived myoblasts in culture. **f,g,** EDB(+) and EDA(+) FN expression analysis in cultured FAPs, MuSC-derived myoblasts, and myotubes, by quantitative PCR. Data was normalized to panFN. **h,** Illustration showing alternative splicing of the mouse FN pre-mRNA with exon 40 coding exon for the IIICS domain. Primers designed for detection of IIICS splicing are indicated by arrows. **i,** Alternative splicing detection of IIICS splicing in MuSC-derived myoblasts by PCR with primers shown in (h). **j,k,** Immunostaining based quantification of EDB(+) FN and EDA(+) FN colocalization with either M-Cad or panFN in *TA* muscle cross sections. **l,m,** Immunostaining for EDB(+) and EDA(+) FN (green) in skeletal muscle cross-sections of young mice 24 hours (h) post injury. CD31 and F4/80 (red) was used to stain endothelial cells and macrophages respectively. DNA (blue) was counterstained using DAPI. White arrowheads show F4/80 positive immune cells. Scale bars = 100 µm (large images) and 20 µm (inserts). Bars represent means ± SEM from cells or tissues of n=4 (a,b) and n=3 (f,g,j,k) biological replicates per condition, each from separate young mice. P values were calculated using a one-tailed Student's *t*-test (a,b,j,k) or one-way ANOVA with Dunnett's (e-g) post-hoc test. $^{**}p<0.01$, $^{***}p<0.001$, $^{****}p<0.0001$. Source data are provided as a Source Data file.

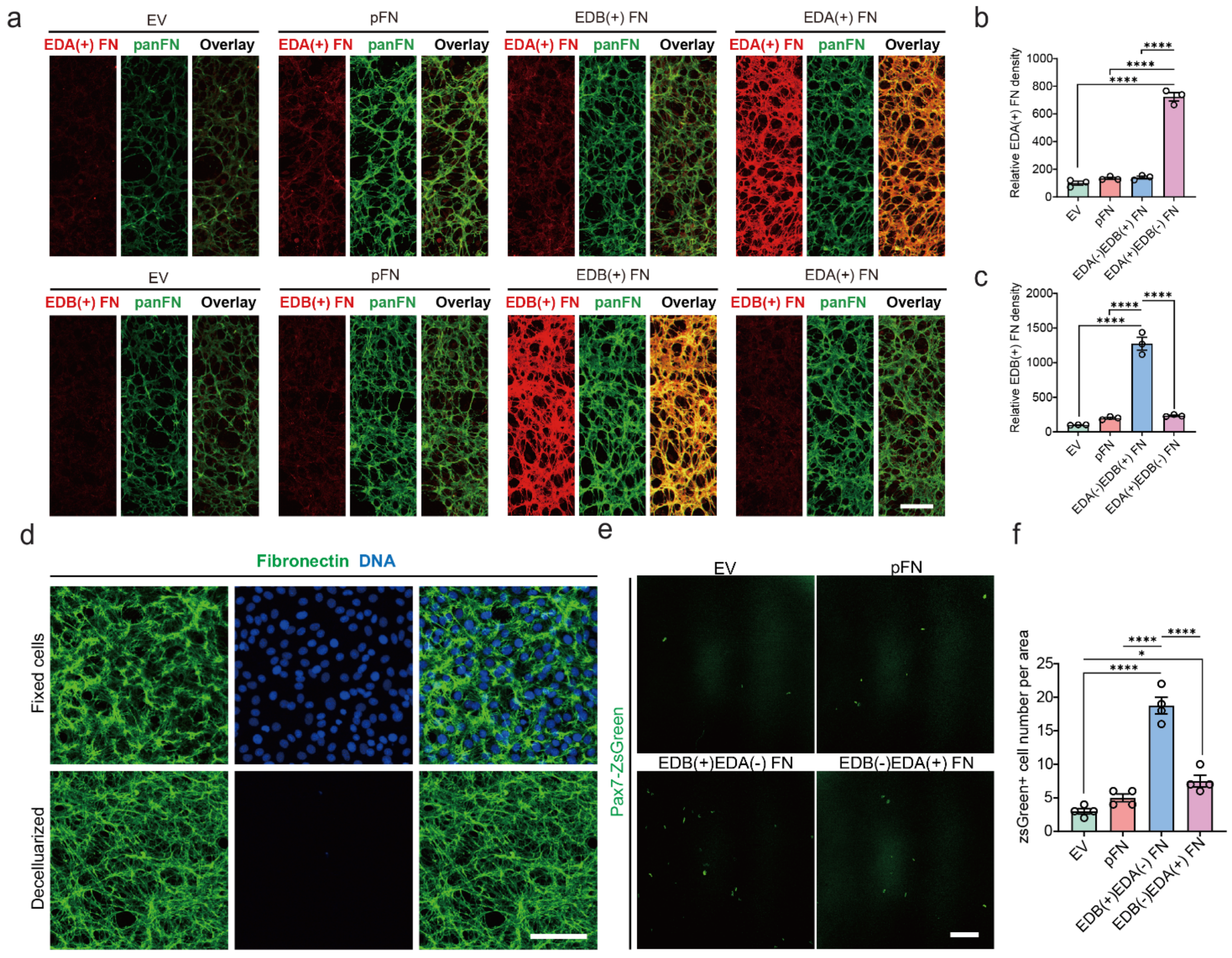

Supplementary Fig. 2: Effect of FN isoforms on myogenic progenitors.

a-c, Representative immunostainings and fluorescence intensity quantification using antibodies specific to the EDB or EDA domains (both red) and panFN (green) in mouse fibroblasts transfected with empty vector (EV), or plasmids expressing pFN (EDB(-)EDA(-) FN), EDB(+)EDA(-) FN, and EDB(-)EDA(+) FN. Scale bar = 100 μm. **d,** Representative panFN (green) immunostaining of mouse fibroblasts with or without decellularization. DNA (blue) indicative of cells was labeled using DAPI. Scale bar = 100 μm. **e,f,** Representative images and quantification of live MuSC-derived Pax7-zsGreen myoblasts after 48 h of culture on ECMs derived from decellularized NIH3T3 mouse fibroblasts transfected with EV, pFN, EDB(+)EDA(-) FN, and EDB(-)EDA(+) FN plasmids. Scale bar = 200 μm. Bars represent means ± SEM from MuSC-derived myoblasts of n=3 (b,c) and n=4 (f) biological replicates per condition, each isolated from separate young mice. P values were calculated using one-way ANOVA with Tukey's (b,c,f) post-hoc test. *$p<0.05$, ****$p<0.0001$. Source data are provided as a Source Data file.

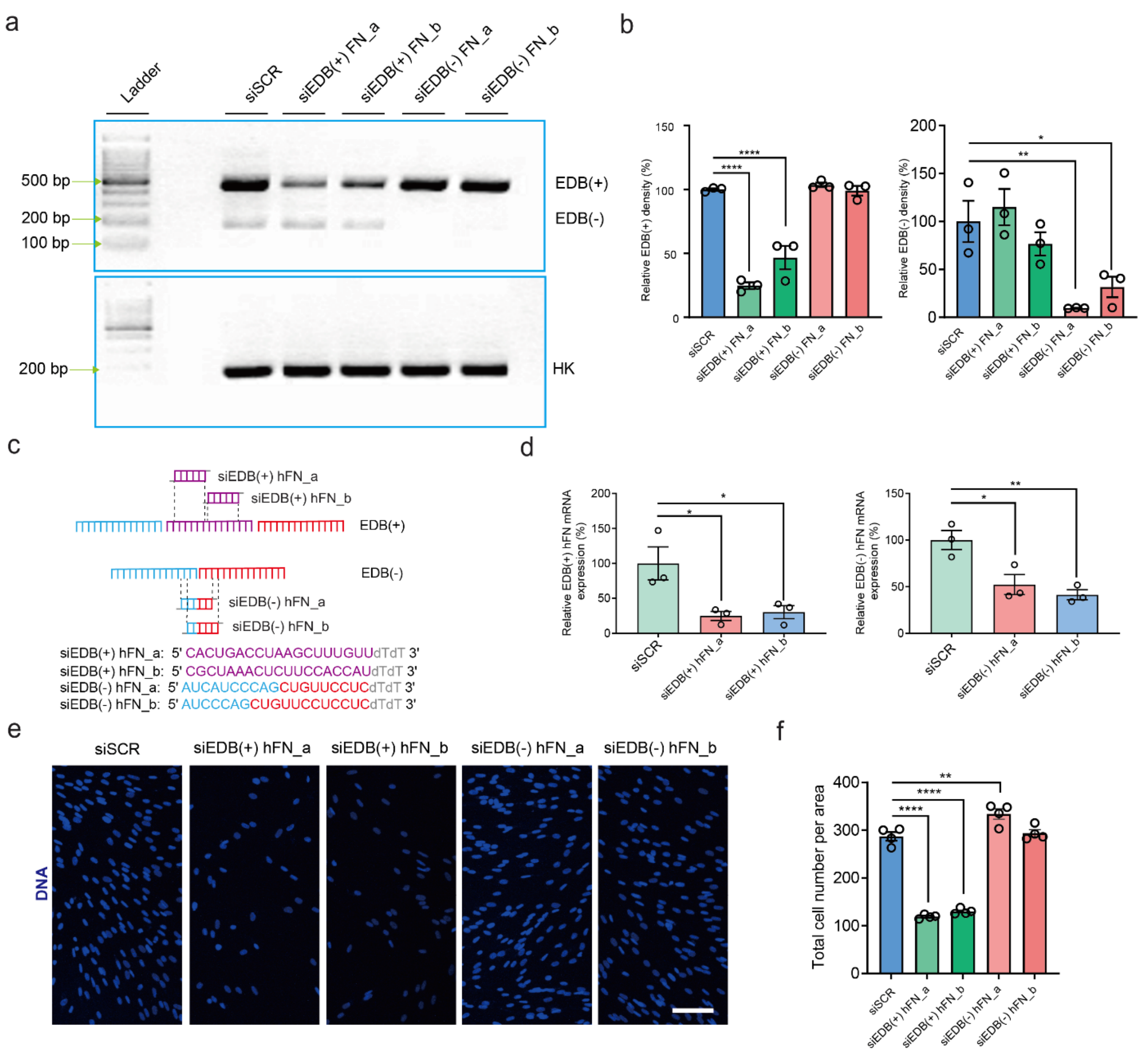

Supplementary Fig. 3: Knockdown of FN splice variants in myogenic progenitors.

a,b, PCR detection and quantification of EDB(+) FN and EDB(-) FN expression in MuSC-derived mouse myoblasts after siRNA treatment. *36b4 (Rplp0)* is shown as housekeeping (HK) gene. **c,** Illustration showing siRNAs targeting human EDB(+) FN (siEDB(+) hFN) or EDB(-) FN (siEDB(-) hFN). Two different siRNAs with the suffix _a or _b were designed for each target. **d,** Quantitative PCR detection of EDB(+) hFN and EDB(-) hFN expression in human MuSC-derived myoblasts after siRNA treatment. The data was normalized to the HK gene *β-actin*. **e,f,** Representative images and quantification of cell numbers after siEDB(+) hFN, siEDB(-) hFN, or siSCR treatment of human MuSC-derived myoblasts based on DNA staining (blue) using DAPI. Scale bar = 100 µm. Bars represent means ± SEM from MuSC-derived myoblasts of n=3 (b,d) and n=4 (f) biological replicates per condition, each from separate young mice or human individuals. P values were calculated using one-way ANOVA with Dunnett's (b,d,f) post-hoc test. *p<0.05, **p<0.01, ****p<0.0001. Source data are provided as a Source Data file.

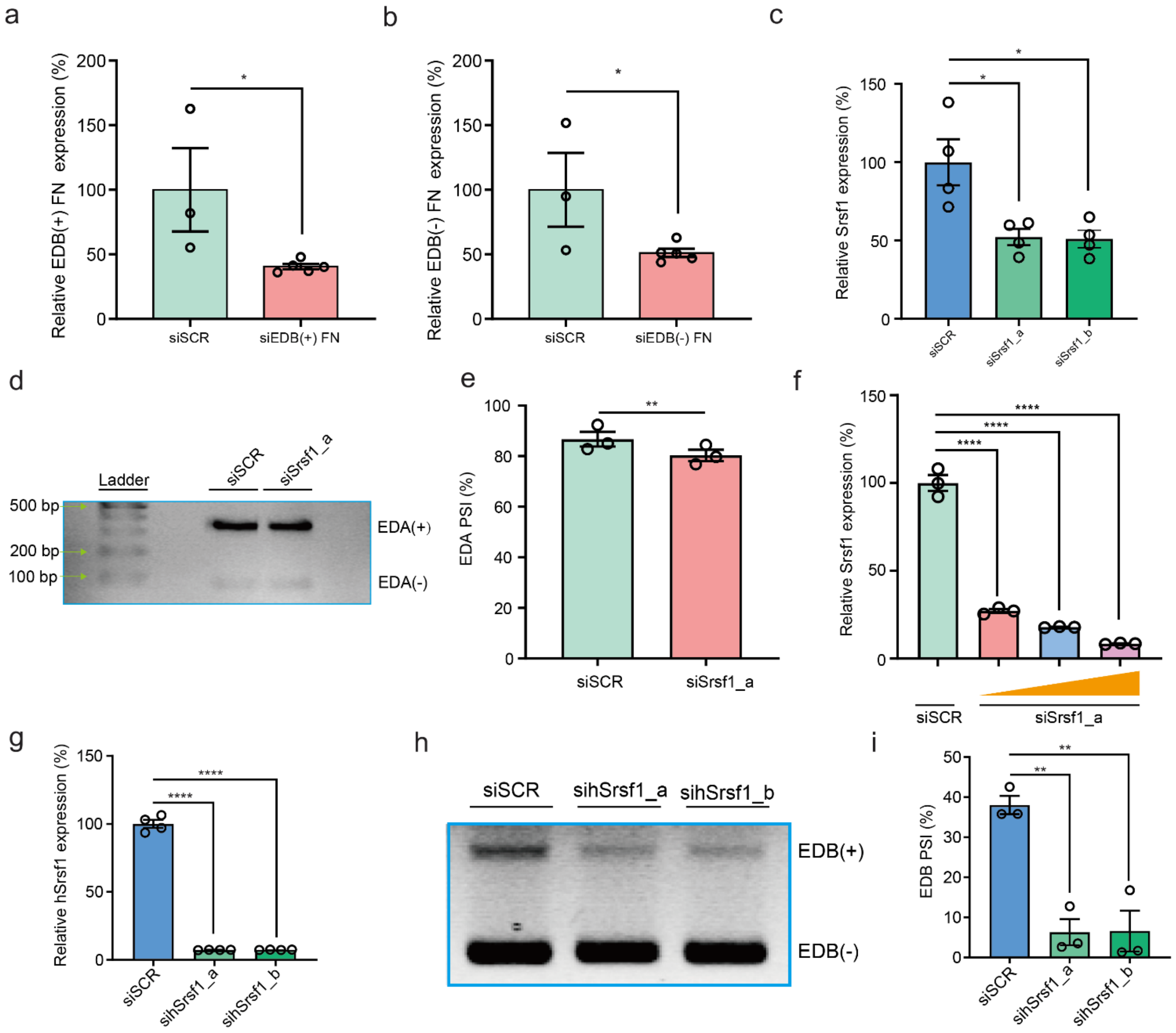
a
Relative EDB(+) FN expression (%)
siSCR
siEDB(+) FN
b
Relative EDB(-) FN expression (%)
siSCR
siEDB(-) FN
c
Relative Srsf1 expression (%)
siSCR
siSrsf1_a
siSrsf1_b
d
Ladder
siSCR
siSrsf1_a
500 bp
200 bp
100 bp
EDA(+)
EDA(-)
e
EDA PSI (%)
siSCR
siSrsf1_a
f
Relative Srsf1 expression (%)
siSCR
siSrsf1_a
g
Relative hSrsf1 expression (%)
siSCR
sihSrsf1_a
sihSrsf1_b
h
siSCR
sihSrsf1_a
sihSrsf1_b
EDB(+)
EDB(-)
i
EDB PSI (%)
siSCR
sihSrsf1_a
sihSrsf1_b

Supplementary Fig. 4: Role of Srsf1 in FN splicing in myogenic progenitors.

a,b, Quantitative PCR of mouse skeletal muscles at 5 dpi treated with isoform specific self-delivering siRNAs targeting EDB(+) FN (siEDB(+) FN (a), EDB(-) FN (siEDB(-) FN (b), compared to the siSCR control. Data was normalized to panFN. Bars represent means ± SEM from tissues of n=3 biological replicates for siSCR treatment and n=5 biological replicates for siEDB(+) or siEDB(-) treatment, each from separate young mice. **c,** Quantitative PCR in MuSC-derived mouse myoblasts after treatment with two different siRNAs targeting Srsf1 (siSrsf1) labeled with the suffix _a or _b. The data was normalized to total RNA. Bars represent means ± SEM from MuSC-derived myoblasts of n=4 biological replicates per condition, each from separate young mice. **d,e** PCR detection and quantification of EDA(+) FN splicing in MuSC-derived mouse myoblasts after siSrsf1_a treatment compared to the siSCR control. The percent spliced in index (PSI) was determined using the formula: in/[in+ex] x 100 (in=exon inclusion, ex=exon exclusion). Bars represent means ± SEM from MuSC-derived myoblasts of n=3 biological replicates per condition, each from separate young mice. **f,** Quantitative PCR of MuSC-derived mouse myoblasts after treatment with increasing levels of siSrsf1_a compared to the siSCR control. *36b4 (Rplp0)* was used as a HK gene. Bars represent means ± SEM from MuSC-derived myoblasts of n=3 biological replicates per condition, each from separate young mice. **g,** Quantitative PCR analysis of MuSC-derived human myoblasts. Human Srsf1 (hSrsf1) was targeted using two different siRNAs (sihSrsf1) labeled with the suffix _a or _b and compared to the siSCR control. The data was normalized to total RNA. Bars represent means ± SEM from MuSC-derived myoblasts of n=4 biological replicates per condition, each from separate human individuals. **h,i**, PCR detection and PSI quantification of EDB(+) hFN splicing in MuSC-derived human myoblasts after sihSrsf1 treatment compared to siSCR. Bars represent means ± SEM from MuSC-derived myoblasts of n=3 biological replicates per condition, each from separate human individuals. P values were calculated using a one-tailed Student's *t*-test (a,b,e) or one-way ANOVA with Dunnett's (c,f,g,i) post-hoc test. *p<0.05, **p<0.01, ****p<0.0001. Source data are provided as a Source Data file.

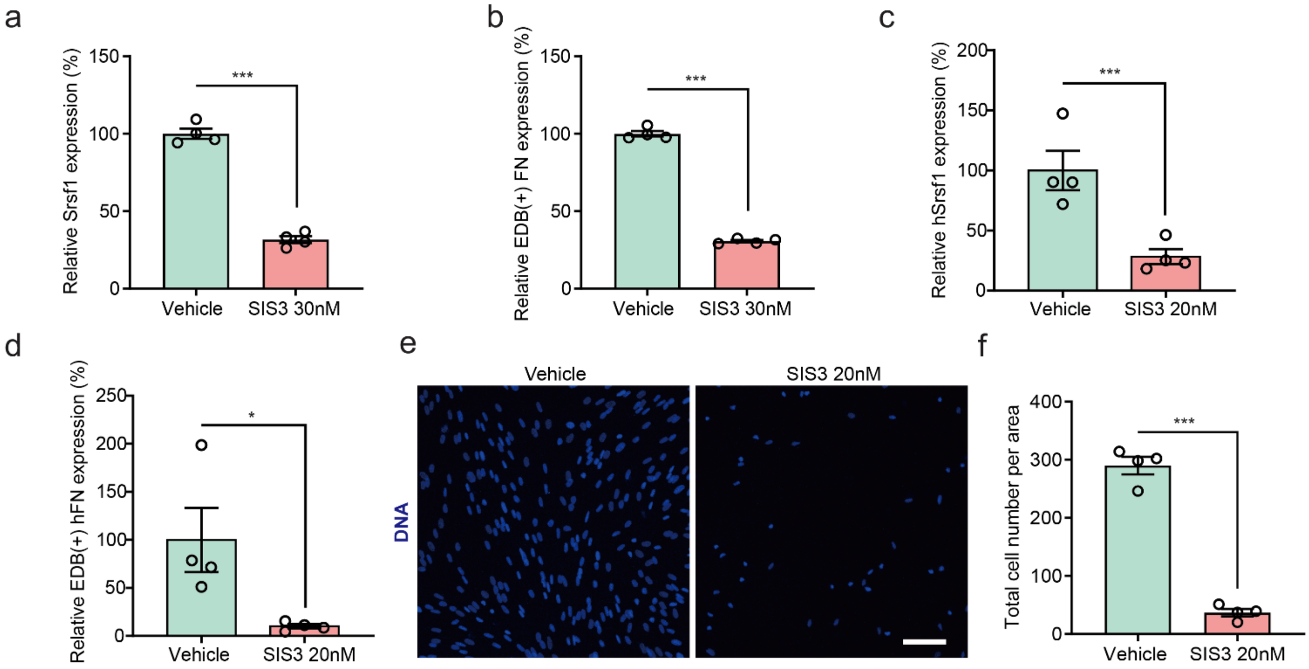

Supplementary Fig. 5: Smad3 controls Srsf1 expression and EDB(+) FN splicing in myogenic progenitors.

a,b, Analysis of *Srsf1* and EDB(+) FN expression by quantitative PCR of MuSC-derived mouse myoblasts after treatment with the Smad3 inhibitor SIS3 compared to the vehicle control. **c,d,** Analysis of hSrsf1 and EDB(+) hFN expression by quantitative PCR of MuSC-derived human myoblasts after treatment with the Smad3 inhibitor SIS3 compared to the vehicle control **e,f,** Representative images and quantification of cell numbers of human MuSC-derived myoblasts after SIS3 treatment compared to the vehicle control based on DNA staining (blue) using DAPI. Scale bar = 100 µm. Bars represent means ± SEM from MuSC-derived myoblasts of n=4 biological replicates per condition, each from separate young mice or human individuals. P values were calculated using a one-tailed Student's *t*-test (a-c,d,f). *p<0.05, ***p<0.001. Source data are provided as a Source Data file.

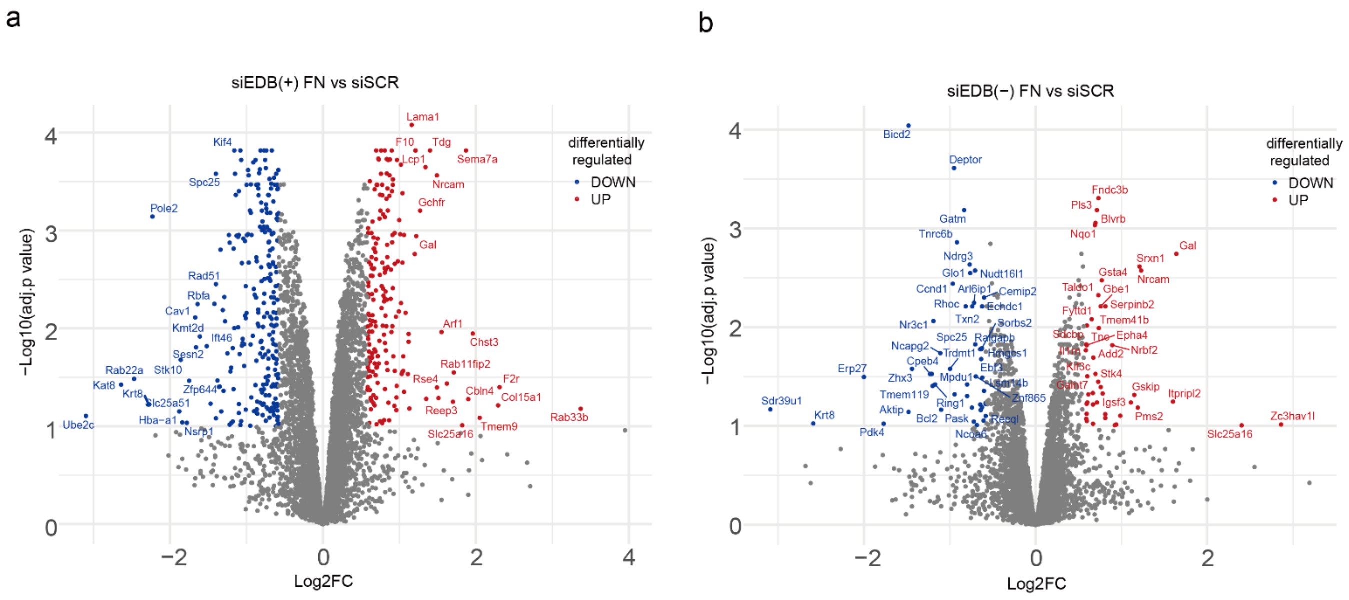

Supplementary Fig. 6: Effect of siEDB(+) FN and siEDB(-) FN on protein abundance in myogenic progenitors.

a,b, Volcano plots showing all quantified proteins after siEDB(+) FN or siEDB(-) FN treatment of MuSC-derived mouse myoblasts. Red dots depict up-regulated proteins and blue dots show down-regulation when compared to the siSCR control. Log2FC > 0.58 and adj p-value < 0.1 were considered as cut-off values (grayed out proteins). Datapoints represent averages from MuSC-derived myoblasts of n=3 young mice per condition.

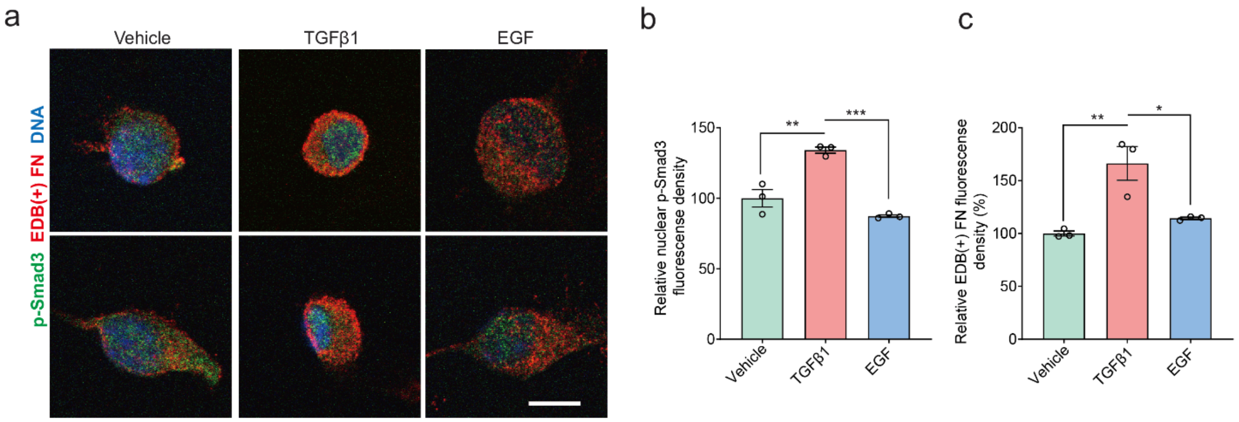

Supplementary Fig. 7: TGFβ1 activates Smad3 signaling and EDB(+) FN splicing in myogenic progenitors.

a-c, Representative images and fluorescence intensity quantification of p-Smad3 (green) and EDB(+) FN (red) staining in MuSC-derived mouse myoblasts treated with TGFβ1, epidermal growth factor (EGF), or the vehicle control. DNA (blue) is counterstained using DAPI. Scale bar = 10 μm. Bars represent means ± SEM from MuSC-derived myoblasts of n=3 biological replicates per condition, each from separate young mice. P values were calculated using one-way ANOVA with Tukey's (b,c) post-hoc test. *p<0.05, **p<0.01, ***p<0.001. Source data are provided as a Source Data file.

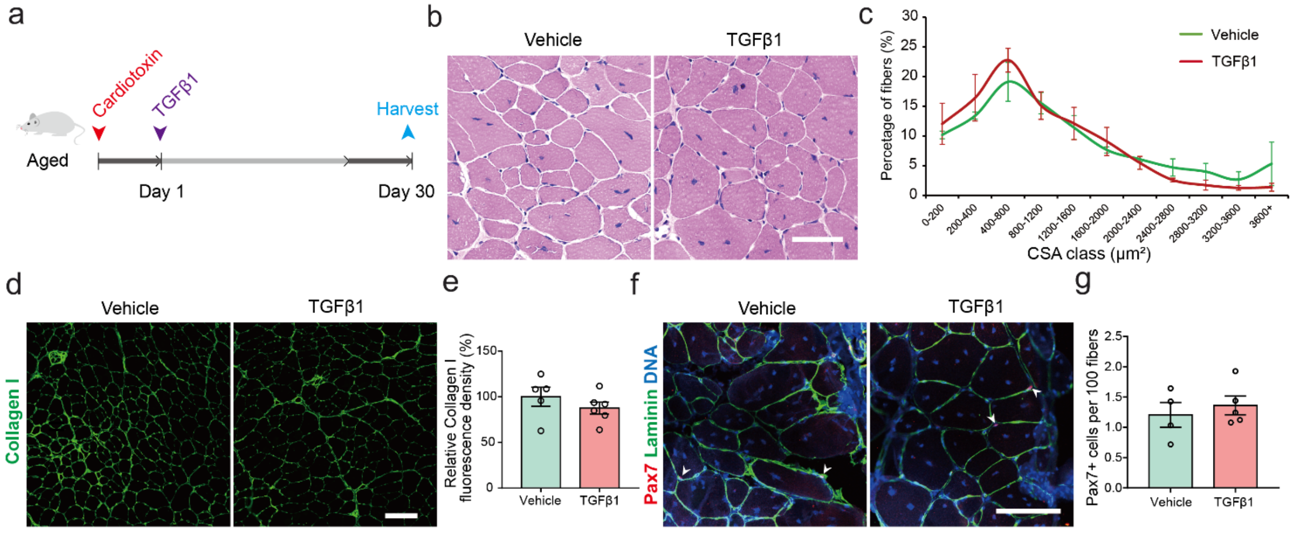

Supplementary Fig. 8: Transient TGFβ1 treatment has no negative long-term effects on aged skeletal muscle.

a, Schematic showing the experimental workflow for TGFβ1 treatment of regenerating muscles of aged mice and sample collection at 30 dpi. **b,** Representative hematoxylin and eosin staining of cross sections of aged mouse TA muscle 30 days after TGFβ1 or vehicle treatment. Scale bar = 50 μm. **c**, CSA quantification of *TA* muscles of aged mice at 30 dpi after treatment with TGFβ1 or vehicle. Datapoints in each CSA class bin represent means ± SEM from n=4 biological replicates for vehicle treatment and n=5 biological replicates for TGFβ1 treatment, each from separate aged mice. **d,e,** Representative immunostaining and quantification of collagen I (green) fluorescence intensity in *TA* muscle cross sections of aged mice at 30 dpi after treatment with TGFβ1 or vehicle. Scale bar = 100 μm. Bars represent means ± SEM from n=5 biological replicates for vehicle treatment and n=6 biological replicates for TGFβ1 treatment, each from separate aged mice. **f,g,** Representative immunostaining and quantification of Pax7+ cells (red, white arrowheads) in cross sections of aged mouse *TA* muscles at 30 dpi after treatment with TGFβ1 or vehicle. The sections were counterstained with laminin and DAPI to visualize DNA (blue). Scale bar = 100 μm. Bars represent means ± SEM from n=4 biological replicates for vehicle treatment and n=5 biological replicates for TGFβ1 treatment, each from separate aged mice. P values were calculated using Student's *t*-test (c,e,g). Source data are provided as a Source Data file.

Supplementary Table 1: siRNAs

Standard custom siRNA, Mouse, Supplier: Dharmacon		
**Primer name**	**Sense**	**Anti-sense**
siSCR	5'-UUCUCCGAACGUGUCACGUdTdT-3'	5'-ACGUGACACGUUCGGAGAAdTdT-3'
siEDB(+) FN_a	5'-CGCUAAACUCUUCCACCAUdTdT-3'	5'-AUGGUGGAAGAGUUUAGCGdTdT-3'
siEDB(+) FN_b	5'-CACUGACCUAAGCUUUGUUdTdT-3'	5'-AACAAAGCUUAGGUCAGUGdTdT-3'
siEDB(-) FN_a	5'-GUCCCAGCUGUCCCUCCUCdTdT-3'	5'-GAGGAGGGACAGCUGGGACdTdT-3'
siEDB(-) FN_b	5'-GUUGUCCCAGCUGUCCCUCdTdT-3'	5'-GAGGGACAGCUGGGACAACdTdT-3'
siSrsf1_a	5'-GAGGCAGGUGAUGUAUGUUdTdT-3'	5'-AACAUACAUCACCUGCCUCdTdT-3'
siSrsf1_b	5'-CUGGCAGGACUUAAAGGAUdTdT-3'	5'-ATCCTTTAAGTCCTGCCAGdTdT-3'
**Self-delivering siRNA_mouse, Accell siRNA, Supplier: Dharmacon**		
siSCR	5'-UUCUCCGAACGUGUCACGUUU-3'	5'-PACGUGACACGUUCGGAGAAUU-3'
siEDB(+) FN	5'-CGCUAAACUCUUCCACCAUUU-3'	5'-PAUGGUGGAAGAGUUUAGCGUU-3'
siEDB(-) FN	5'-GUCCCAGCUGUCCCUCCUCUU-3'	5'-PGAGGAGGGACAGCUGGGACUU-3'
**Standard custom siRNA, Human, Supplier: Dharmacon**		
sihSrsf1_a	5’-AGGACAUUGAGGACGUGUUdTdT-3’	5'-AACACGUCCUCAAUGUCCUdTdT-3'
sihSrsf1_b	5’-AGUGGAAGUUGGCAGGAUUdTdT-3’	5'-AAUCCUGCCAACUUCCACUdTdT-3'
siEDB(+) hFN_a	5'-CGCUAAACUCUUCCACCAUdTdT-3'	5'-AUGGUGGAAGAGUUUAGCGdTdT-3'
siEDB(+) hFN_b	5'-CACUGACCUAAGCUUUGUUdTdT-3'	5'-AACAAAGCUUAGGUCAGUGdTdT-3'
sihEDB(-) hFN_a	5'-AUCAUCCCAGCUGUUCCUCdTdT-3'	5'-GAGGAACAGCUGGGAUGAUdTdT-3'
sihEDB(-) hFN_b	5'-AUCCCAGCUGUUCCUCCUCdTdT-3'	5'-GAGGAGGAACAGCUGGGAUdTdT-3'

Supplementary Table 2: Antibodies

Antibody	Cat.NO/format	Supplier	Host Species/isotype	Application	Dilution
act-Integrin β1	553715	BD Bioscience	Rat, IgG2a	IF	1:100
CD31	13031982	Invitrogen	Mouse, IgG1	IF	1:100
CD56	BDB555515	Thermo Scientific	Mouse, IgG1	MACS	1:200
Collagen I	ab34710	Abcam	Rabbit, IgG	IF	1:100
Control IgG	30000-0-AP	ProteinTech	Rabbit, polyclonal IgG	ChIP	1:100
EDA(+) FN	ab6328	Abcam	Mouse, IgG1	IF	1:100
EDB(+) FN	ab154210	Abcam	Mouse, IgG1	IF	1:100
eMyHC	Supernatant	Developmental Studies Hybridoma Bank	Mouse, IgG1	IF	1:10
F4/80	MCA497GA	Bio-Rad	Rat, IgG2b	IF	1:100
ki67	ab833	Abcam	Rabbit, polyclonal IgG	IF	1:200
Laminin	L9393	Sigma Aldrich	Rabbit, polyclonal IgG	IF	1:200
Laminin-α2	L0663	Sigma Aldrich	Rat, IgG1	IF	1:200
M-Cadherin	D4B9L	Cell Signaling Technology	Rabbit, IgG	IF	1:100
panFN	F3648	Sigma Aldrich	Rabbit, polyclonal IgG	IF	1:100
Pax7	Concentrated	Developmental Studies Hybridoma Bank	Mouse, IgG1	IF	1:100
PDGFRα	BAF1062	R&D Technologies	Goat, polyclonal IgG	MACS	1:200
p-Smad1/5/9	13820	Cell Signaling Technology	Rabbit, IgG	ChIP	1:100
p-Smad3	9520	Cell Signaling Technology	Rabbit, IgG	IF/ChIP	1:100
Srsf1	MABE163	EMD Millipore	Mouse, IgG2b	IF	1:100
Vcam1	105704	Biolegend	Rat, IgG2a	MACS	1:200

Supplementary Table 3: Plasmids

Plasmid name	Addgene ID
7iBi89 (Rat fibronectin EDB minigene plasmid)	Addgene Plasmid #14065
pEGFP×3-N1	Addgene Plasmid #86776
pCDNA3.1_3XFlag	Addgene Plasmid #182494
pcDNA-FLAG-SF2	Addgene Plasmid #99021
Fibronectin-human-plasma in pMAX	Addgene Plasmid #120401
Fibronectin-human-EDA in pMAX	Addgene Plasmid #120402
Fibronectin-human-EDB in pMAX	Addgene Plasmid #120403

Supplementary Table 4: Chemicals and Growth factors

Chemicals & Growth factors	Supplier	Cat.NO
Recombinant Mouse TGFβ1	Biolegend	763102
Cardiotoxin (CTX)	Latoxan	L8102
SIS3 (Smad3 inhibitor)	Sigma Aldrich	566405
Recombinant Human bFGF Protein	R&D Technologies	3139-FB-025
Recombinant Human EGF Protein	R&D Technologies	2028-EG
Tamoxifen	Toronto Research Chemicals	T006000-25

Supplementary Table 5: Primers

Primers, Supplier: Integrated DNA Technologies	
**Primer name**	Sequence
FN-E25_Forward	TGCCCCTATCTCTGATACCG
FN-E25_Reverse	AGCTCTGCAACGTCCTCTTC
FN-E33_Forward	GGTTCAAACTGCAGTGACCA
FN-E33_Reverse	GCAGTAAAGCTGGTGGGTGT
FN-E40_Forward	ACGTCATTGCCCTGAAGAAC
FN-E40_Reverse	AACCTCTTCCCGAACCTTGT
FN-E25-E33-Forward_1	GGGGACCTCTCTGGAAGAAG
FN-E25-E33-Reverse_1	GCAGTAAAGCTGGTGGGTGT
FN-E25-E33-Forward_2	GAATCCTGGCCTGGAGTACA
FN-E25-E33-Reverse_2	CACTGGGCAGTAAAGCTGGT
Rplp0_Forward	TAAAGACTGGAGACAAGGTGGGAG
Rplp0_Reverse	AGAAAGCGAGAGTGCAGGGC
Srsf1_Forward	CGCATCTACGTGGGTAACCT
Srsf1_Reverse	TAGCCGTCGTAGTCGTAGCC
ChIP_Forward-1	ATAAAGGCGCCATTTTGGAG
ChIP_Reverse-1	AAGAGGCCTTCCTACCAAGC
ChIP_Forward-2	ATAAAGGCGCCATTTTGGAG
ChIP_Reverse-2	AGGCCTTCCTACCAAGCCTA
ChIP_Forward-3	TGCTTCTCAGCACCCGTTAG
ChIP_Reverse-3	CTCCAAAATGGCGCCTTTAT
EDB(+) FN _Forward	CCCAGCTCACTGACCTAAGC
EDB(+) FN_Reverse	ATAGTCAATGCCAGGCTCCA
EDB(-) FN _Forward	TACCGTTGTCCCAGCTGTCC
EDB(-) FN _Reverse	CCCAGGCAGGAGATTTGTTA
EDA(+) FN_Forward	CCCTAAAGGACTGGCATTCA
EDA(+) FN_Reverse	TCTGCAGTGTCGTCTTCACC
panFN_Forward	AATGGAAAAGGGGAATGGAC
panFN_Reverse	CTCGGTTGTCCTTCTTGCTC
miniG-vec-Forward	TGCCCCTATCTCTGATACCG
miniG-exon-Reverse	GCCCCTTGCTCCTACCAC
hSrsf1-Forward	TACCTCCAGACATCCGAACC
hSrsf1_Reverse	GCAGACGGTACCCATCGTAA
hFN-E25-Forward	CAGCAGGGAAATTCTTTGGA
hFN-E25_Reverse	AATGTTGGTGAATCGCAGGT
hActin-Forward	GATCATTGCTCCTCCTGAGC
hActin_Reverse	ACTCCTGCTTGCTGATCCAC
EDB(+) hFN _Forward	GCCCCAACTCACTGACCTAA
EDB(+) hFN_Reverse	TCCAGCCCTGTGACTGTGTA
