# Supplementary material for: TGFβ-Smad3 signaling restores cell-autonomous Srsf1-mediated splicing of fibronectin in aged skeletal muscle stem cells
Source: Nat Commun. 2025 Nov 21;16:11532. doi: 10.1038/s41467-025-66582-2 (PMC12748718; doi:10.1038/s41467-025-66582-2)
Supplement: Supplementary file 2 — Reporting Summary [file 41467_2025_66582_MOESM2_ESM.pdf]

nature portfolio

Corresponding author(s): C. Florian Bentzinger

Last updated by author(s): 25-08-2025

Reporting Summary

Nature Portfolio wishes to improve the reproducibility of the work that we publish. This form provides structure for consistency and transparency in reporting. For further information on Nature Portfolio policies, see our Editorial Policies and the Editorial Policy Checklist.

Statistics

For all statistical analyses, confirm that the following items are present in the figure legend, table legend, main text, or Methods section.

n/a	Confirmed	
☐	☒	The exact sample size (*n*) for each experimental group/condition, given as a discrete number and unit of measurement
☐	☒	A statement on whether measurements were taken from distinct samples or whether the same sample was measured repeatedly
☐	☒	The statistical test(s) used AND whether they are one- or two-sided<br>*Only common tests should be described solely by name; describe more complex techniques in the Methods section.*
☒	☐	A description of all covariates tested
☐	☒	A description of any assumptions or corrections, such as tests of normality and adjustment for multiple comparisons
☐	☒	A full description of the statistical parameters including central tendency (e.g. means) or other basic estimates (e.g. regression coefficient) AND variation (e.g. standard deviation) or associated estimates of uncertainty (e.g. confidence intervals)
☐	☒	For null hypothesis testing, the test statistic (e.g. *F*, *t*, *r*) with confidence intervals, effect sizes, degrees of freedom and *P* value noted<br>*Give P values as exact values whenever suitable.*
☒	☐	For Bayesian analysis, information on the choice of priors and Markov chain Monte Carlo settings
☒	☐	For hierarchical and complex designs, identification of the appropriate level for tests and full reporting of outcomes
☒	☐	Estimates of effect sizes (e.g. Cohen's *d*, Pearson's *r*), indicating how they were calculated

Our web collection on statistics for biologists contains articles on many of the points above.

Software and code

Policy information about availability of computer code

Data collection	Transcription factor binding sites in the Srsf1 promoter were identified using Pscan (http://159.149.160.88/pscan/).
Data analysis	Gene expression matrices were analyzed using Seurat Version 4.3.026. Gene expression density was generated with Nebulosa version 1.8.0.3. Molecular modeling was performed using the MOE software (Chemical Computing Group, version MOE 2024.06). Mass spectrometry data was analyzed using DIA-NN software (version 1.9.2) and R studio 2022.07.2+576. Images were quantified using ImageJ (version 1.53t). Statistical analysis was performed using GraphPad Prism (GraphPad Software, version 9.4.1 (681)).

For manuscripts utilizing custom algorithms or software that are central to the research but not yet described in published literature, software must be made available to editors and reviewers. We strongly encourage code deposition in a community repository (e.g. GitHub). See the Nature Portfolio guidelines for submitting code & software for further information.

Data

Policy information about availability of data

All manuscripts must include a data availability statement. This statement should provide the following information, where applicable:

- Accession codes, unique identifiers, or web links for publicly available datasets
- A description of any restrictions on data availability
- For clinical datasets or third party data, please ensure that the statement adheres to our policy

No new code was generated in the context of this publication. The single-cell sequencing dataset analysed in this study is available in Gene Expression Omnibus (GEO, https://www.ncbi.nlm.nih.gov/geo/) under the accession number GSE143437 [https://www.ncbi.nlm.nih.gov/geo/query/acc.cgi?acc=GSE143437]. Mass

spectrometry proteomics raw files and the respective data analysis files have been deposited to the ProteomeXchange Consortium (http://proteomecentral.proteomexchange.org) via the PRIDE partner repository with identifier PXD062037 [http://proteomecentral.proteomexchange.org/cgi/GetDataset?ID=PXD062037]. The structure of human Srsf1 is available in RCSB Protein Data Bank (https://www.rcsb.org/) under structure number 2M8D [https://www.rcsb.org/structure/2M8D]. Source data are provided with this paper.

Research involving human participants, their data, or biological material

Policy information about studies with human participants or human data. See also policy information about sex, gender (identity/presentation), and sexual orientation and race, ethnicity and racism.

Reporting on sex and gender	Human tissues were a by-product of orthopedic knee surgeries collected after informed consent, which included anonymized reporting of biological sex and age. Tissues from 3 male and 1 female donor aged between 14 and 26 years were used to generate 4 batches of primary human myoblasts. No differences in the characteristics of primary myoblast batches derived from male or female tissue were observed. Therefore, samples/datapoints were pooled and considered equal biological replicates.
Reporting on race, ethnicity, or other socially relevant groupings	N/A
Population characteristics	N/A
Recruitment	Biological sex and age of donors were dictated by scheduled surgical procedures.
Ethics oversight	Collection was approved by the bureau d'autorisation des projets de recherche (BAPR) CIUSSS de l'Estrie – CHUS, Université de Sherbrooke (ethical protocol 2019-2608).

Note that full information on the approval of the study protocol must also be provided in the manuscript.

Field-specific reporting

Please select the one below that is the best fit for your research. If you are not sure, read the appropriate sections before making your selection.

[x] Life sciences [ ] Behavioural & social sciences [ ] Ecological, evolutionary & environmental sciences

For a reference copy of the document with all sections, see nature.com/documents/nr-reporting-summary-flat.pdf

Life sciences study design

All studies must disclose on these points even when the disclosure is negative.

Sample size	Sample size determination was based on the expected effect size and variability that was previously observed for similar readouts in the investigator's laboratory.
Data exclusions	No data was excluded.
Replication	All findings reported in the manuscript were successfully reproduced. Reproducibility was ensured through independent biological replicates from cells or tissues of separate mice or humans, with the number of replicates indicating the independent repetitions as specified in the figure legends. No results were excluded due to lack of reproducibility.
Randomization	Animals were randomized by body weight within their experimental groups. For human myoblasts, randomization was not applicable. Independent donor samples served as biological replicates to ensure reproducibility.
Blinding	In vivo treatments were not blinded. However, investigators were blinded to group allocation during both data collection and analysis.

Reporting for specific materials, systems and methods

We require information from authors about some types of materials, experimental systems and methods used in many studies. Here, indicate whether each material, system or method listed is relevant to your study. If you are not sure if a list item applies to your research, read the appropriate section before selecting a response.

Materials & experimental systems

n/a	Involved in the study
☐	☒ Antibodies
☐	☒ Eukaryotic cell lines
☒	☐ Palaeontology and archaeology
☐	☒ Animals and other organisms
☒	☐ Clinical data
☒	☐ Dual use research of concern
☒	☐ Plants

Methods

n/a	Involved in the study
☒	☐ ChIP-seq
☒	☐ Flow cytometry
☒	☐ MRI-based neuroimaging

Antibodies

Antibodies used

Antibodies were reported in Supplementary Table 2

act-Integrin β1, 553715, BD Bioscience, dilution: 1:100
CD31, 13031982, Invitrogen, dilution: 1:100
CD56, BDB555515, Thermo Scientific, dilution: 1:200
Collagen I, ab34710, Abcam, dilution: 1:100
Control IgG, 30000-0-AP, ProteinTech, dilution: 1:100
EDA(+) FN, ab6328, Abcam, dilution: 1:100
EDB(+) FN, ab154210, Abcam, dilution: 1:100
eMyHC, Supernatant, Developmental Studies Hybridoma Bank, dilution: 1:10
F4/80, MCA497GA, Bio-Rad, dilution: 1:100
ki67, ab833, Abcam, dilution: 1:200
Laminin, L9393, Sigma Aldrich, dilution: 1:200
Laminin-α2, L0663, Sigma Aldrich, dilution: 1:200
M-Cadherin, D4B9L, Cell Signaling Technology, dilution: 1:100
panFN, F3648, Sigma Aldrich, dilution: 1:100
Pax7, Concentrated, Developmental Studies Hybridoma Bank, dilution: 1:100
PDGFRα, BAF1062, R&D Technologies, dilution: 1:200
p-Smad1/5/9, 13820, Cell Signaling Technology, dilution: 1:100
p-Smad3, 9520, Cell Signaling Technology, dilution: 1:100
Srsf1, MABE163, EMD Millipore, dilution: 1:100
Vcam1, 105704, Biolegend, dilution: 1:200

Validation

1. Pax7 (Pax7, DHSB): This antibody has been deposited to the Developmental Studies Hybridoma Bank by Kawakami, A. Multiple publications describe this antibody. Selected reference: Lai et al., Nature, 2024. DOI: 10.1038/s41586-024-07348-6

2. panFN (F3648, Sigma Aldrich): This antibody has been validated by the manufacturer by Immunofluorescence in human HS-68 cells. Multiple publications describe this antibody. Selected reference: Curnow et al., PLOS one, 2010. DOI: 10.1371/journal.pone.0009730

3. EDB(+) FN (ab154210, Abcam): This antibody has been validated by the manufacturer by immunohistochemistry analysis of human glioblastoma frozen sections. Multiple publications describe this antibody. Selected reference: Carnemolla et al., J Biol Chem,1992. DOI: 10.1016/S0021-9258(18)35819-8

4. EDA(+) FN (ab6328, Abcam): This antibody has been validated by the manufacturer by immunohistochemistry with Formalin fixed paraffin embedded rat kidney tissue. Multiple publications describe this antibody. Selected reference: Brizio et al., Stem Cell Res Ther, 2024. DOI: 10.1186/s13287-024-03916-9

5, M-Cadherin (D4B9L, Cell Signaling Technology): This antibody has been validated by the manufacturer by immunofluorescence with skeletal muscle fiber staining. Multiple publications describe this antibody. Selected reference: Schüler et al., Curr Protoc, 2021. DOI: 10.1002/cpz1.263

6, ki67 (ab833, Abcam): This antibody has been validated by the manufacturer by immunofluorescence with human skin tissue. Multiple publications describe this antibody. Selected reference: Li et al., J Transl Med, 2023. DOI: 10.1186/s12967-023-04190-8

7. eMyHC (F1.652, DSHB): This antibody has been deposited to the Developmental Studies Hybridoma Bank by Blau, H.M. and has been validated by immunofluorescence with mouse quadriceps muscle tissue. Multiple publications describe this antibody. Selected reference: Lukjanenko et al., Nat Med, 2017. DOI:10.1038/nm.4126

8. Laminin-α2 (L0663, Sigma Aldrich): This antibody has been validated by the manufacturer by immunofluorescence with mouse tongue tissue. Multiple publications describe this antibody. Selected reference: Liu et al., Stem cell reports, 2020. DOI: 10.1016/j.stemcr.2020.08.004

9. Laminin (L9393, Sigma Aldrich): This antibody has been validated by the manufacturer by immunofluorescence with human tongue tissue. Multiple publications describe this antibody. Selected reference: Ham et al., Nat Commun, 2025. DOI: 10.1038/s41467-025-58542-7

10. Srsf1 (MABE163, EMD Millipore): This antibody has been validated by the manufacturer by immunohistochemistry in human prostate carcinoma cells and human testis tissues. Multiple publications describe this antibody. Selected reference: Sridhara et al., Cell Rep, 2017. DOI: 10.1016/j.celrep.2016.12.050

11. p-Smad3 (9520, Cell Signaling Technology): This antibody has been validated by the manufacturer by Chromatin immunoprecipitations with TGFβ3 treated HaCaT cells. Multiple publications describe this antibody. Selected reference: Yang et al., Cell Death Dis, 2019. DOI: 10.1038/s41419-019-1797-5

12. p-Smad1/5/9 (13820, Cell Signaling Technology): This antibody has been validated by the manufacturer by Chromatin immunoprecipitations with BMP treated MCF7 cells. Multiple publications describe this antibody. Selected reference: Palacio et al., Nat Commun, 2025. DOI: 10.1038/s41467-025-56221-1

13. act-Integrin β1 (553715, BD Bioscience): Multiple publications describe this antibody.
Selected reference: Molè et al., Dev Cell, 2020. DOI: 10.1016/j.devcel.2020.01.012

14. Collagen I (ab34710, Abcam): This antibody has been validated by the manufacturer by immunohistochemistry analysis of human stomach mucosa tissue. Multiple publications describe this antibody. Selected reference: He et al., Nat Commun, 2025. DOI: 10.1038/s41467-025-58074-0

15. CD31 (13031982, Invitrogen): Multiple publications describe this antibody. Selected reference: Le Moal et al., Sci Transl Med, 2024. DOI: 10.1126/scitranslmed.abn8529

16. F4/80 (MCA497GA, Bio-Rad): Multiple publications describe this antibody. Selected reference: Li et al., Front Physiol,2025. DOI: 10.3389/fphys.2025.1534911

17. Control IgG (30000-0-AP, ProteinTech): This antibody has been validated by the manufacturer using different secondary antibodies. Multiple publications describe this antibody. Selected reference: Tong et al., Cell Metabolism, 2024. DOI: 10.1016/j.cmet.2024.10.019

18. PDGFRα (BAF1062, R&D Technologies): This antibody has been validated by the manufacturer by immunohistochemistry using mouse embryos. Multiple publications describe this antibody. Selected reference: Schüler et al., Curr Protoc, 2021. DOI: 10.1002/cpz1.263

19. CD56 (BDB555515, Thermo Scientific): Multiple publications describe this antibody. Selected reference: : Le Moal et al., Sci Transl Med, 2024. DOI: 10.1126/scitranslmed.abn8529

20. Vcam1 (105704, Biolegend): This antibody has been validated by the manufacturer by Flow cytometry using C57BL/6 bone marrow myeloid cells. Multiple publications describe this antibody. Selected reference: Sarshad et al., Mol Cell, 2018, DOI: 10.1016/j.molcel.2018.07.020

Eukaryotic cell lines

Policy information about cell lines and Sex and Gender in Research

Cell line source(s)	NIH-3T3 mouse fibroblasts were obtained from Signosis (PC-004). Primary mouse myoblasts were obtained from C57BL/6 mice (JAX mice, strain #:000664). For all experiments with primary mouse myoblasts, only freshly isolated cells, or cells cultured to a maximum of 2 passages, obtained from male and female mice at equal proportions, were used. Primary human myoblasts were obtained from 3 male and 1 female donor. Human myoblasts were cultured up to a maximum of 4 passages.
Authentication	NIH-3T3 mouse fibroblasts were authenticated based on fibroblast morphology and the expression of mouse housekeeper genes. Primary myoblasts were authenticated based on morphology, mouse or human housekeeper expression, and their ability to fuse into multinucleated myotubes.
Mycoplasma contamination	NIH-3T3 mouse fibroblasts and primary cells tested negative for mycoplasma.
Commonly misidentified lines (See ICLAC register)	N/A

Animals and other research organisms

Policy information about studies involving animals; ARRIVE guidelines recommended for reporting animal research, and Sex and Gender in Research

Laboratory animals	6-10 week-old young and 22-25 month-old aged C57BL/6 mice were purchased from JAX mice (strain #:000664) or the Quebec Network of Research on Ageing (RQRV).
Wild animals	The study did not involve wild animals.

Reporting on sex	Experiments were designed to include male and female mice in approximately equal proportions, and animals were randomized into experimental groups. Data was disaggregated for sex but no difference were observed in any of the experiments. Therefore, samples/datapoints were pooled and considered equal biological replicates.
Field-collected samples	The study did not involve field-collected samples.
Ethics oversight	Husbandry and all experimental protocols using mice were performed in accordance with the guidelines established by the animal committee of the Université de Sherbrooke, which are based on the guidelines of the Canadian Council on Animal Care (ethical protocol 2022-3553).

Note that full information on the approval of the study protocol must also be provided in the manuscript.

Plants

Seed stocks	N/A
Novel plant genotypes	N/A
Authentication	N/A
